# Plasmacytoid Dendritic Cells Protect Against Middle Cerebral Artery Occlusion Induced Brain Injury by Priming Regulatory T Cells

**DOI:** 10.3389/fncel.2020.00008

**Published:** 2020-01-31

**Authors:** Chen Chen, Zhang Chencheng, Liu Cuiying, Geng Xiaokun

**Affiliations:** China-America Institute of Neuroscience, Beijing Luhe Hospital, Capital Medical University, Beijing, China

**Keywords:** plasmacytoid dendritic cells (pDCs), regulatory T cells (Tregs), middle cerebral artery occlusion (MCAO), indoleamine 2, 3-dioxygenase 1 (IDO1), inflammation

## Abstract

Regulatory T cells (Tregs) play an anti-inflammatory effect to protect against ischemic stroke. Plasmacytoid dendritic cells (pDCs) can induce regulatory T cells tolerance in sterile-inflammation conditions. However, whether and how pDCs-mediated Tregs response play a part in the pathology of ischemic stroke remains unclear. In this study, we showed that pDCs were increased in the brain of middle cerebral artery occlusion (MCAO) mice. Depletion of pDCs with 120G8 exacerbated MCAO-induced brain injury, peripheral pro-inflammation response and decreased the systemic Tregs in mice. Furthermore, the data of mixed lymphocyte reaction (MLR) *in vitro* demonstrate that splenic pDCs from MCAO mice can significantly promote Tregs proliferation, accompanying with the increased expression of indoleamine 2,3-dioxygenase 1 (IDO1) on pDCs. Taken together, the findings here suggested that under the pathologic state of stroke, pDCs protect against MCAO-induced brain injury by priming Tregs, illustrating that pDCs represented as a therapeutic target for the prevention of ischemic brain injury.

## Introduction

Stroke is the leading cause of lethality and permanent disability throughout the world (Feigin, 2019). Presently, although common approaches, such as medical thrombolysis and mechanical thrombectomy, have been applied to treat stroke, delayed thrombolytic treatment with recombinant tissue plasminogen activator (tPA) and poor outcomes still occur. Thereby, it is urgent to identify the pathology of stroke and develop a promising therapeutic target for effective treatment against stroke. Recent studies demonstrate that immune response, especially mediated by T lymphocytes plays an important role in the pathology of stroke (Vidale et al., 2017; Shekhar et al., 2018; Nakamura and Shichita, 2019). Among T lymphocytes subsets, accumulating evidence indicates regulatory T cells (Tregs), which function to suppress excessive immune responses, play an anti-inflammatory effect to protect against ischemic stroke (Duffy et al., 2018). Previous studies have shown that adoptive Tregs therapy could ameliorate blood-brain barrier damage after cerebral ischemia and TPA-induced brain hemorrhage after stroke (Li et al., 2013; Li P. et al., 2017; Mao et al., 2017). The latest clinical data show that a significant reduction of peripheral Tregs frequency were observed in patients with ischemic stroke compared with controls (Dolati et al., 2018), which indicates that Tregs participate in the pathology of stroke by migrating into the brain. However, how the Tregs were primed on the stroke progression has not been fully illustrated.

Dendritic cells (DCs) are key actors when an adaptive immune response is initiated. There are two major DCs subsets in mice, classical DCs (cDCs) and plasmacytoid dendritic cells (pDCs), which are characterized by differential expression of cell surface markers and functions. cDCs express CD11c^hi^B220^−^ and highly specialized in antigen presentation. The pDCs express CD11c^int^CD317^+^ and are essential for an antiviral response (Segura, 2016). The pDCs also have the ability to induce tolerogenic immune responses in many types of human cancers and autoimmune/inflammatory diseases (Lombardi et al., 2015; Swiecki and Colonna, 2015). These tolerogenic properties of pDCs are mostly associated with the induction of Tregs (Li S. et al., 2017). For example, the resident pDCs inhibit cytotoxic T cell function and directly favor melanoma growth by triggering IL-10 secreting Tregs (Stubbe et al., 2013). The tumor-associated pDCs contribute to breast cancer progression by sustaining the expansion of Tregs (Sisirak et al., 2012). Gilliet and collegues found that in ovarian cancer the interaction of ICOSL+pDCs and ICOS+ Tregs can lead the tumor progression (Conrad et al., 2012). In type I diabetes, the interactions between pDCs and iNKT cells induce the T cells to differentiate into Tregs in pancreatic lymph nodes (Diana et al., 2013). In atherosclerosis, though many reports have provided conflicting evidence for pDCs’ role in mouse models (Daissormont et al., 2011; Döring et al., 2012; Macritchie et al., 2012; Sage et al., 2014), the most recent study showed that IDO1+ aortic pDCs could protect against atherosclerosis by induction of Tregs (Yun et al., 2016). Nevertheless, the effect of pDCs on ischemic stroke and underlying mechanism remain unclear.

In this study, we attempted to explore the role of pDCs and the underlying mechanism in ischemic stroke progression. First, we showed that pDCs were increased in the brain of middle cerebral artery occlusion (MCAO) mice. Depletion of pDCs exacerbates MCAO-induced brain injury, peripheral pro-inflammation response and decreases the systemic Tregs in mice. Furthermore, the data of mixed lymphocyte reaction (MLR) demonstrate that splenic pDCs from MCAO mice can significantly promote Tregs proliferation *in vitro*, accompanying with the increased expression of indoleamine 2,3-dioxygenase 1 (IDO1) on pDCs. Collectively, our data here emphasized the neuroprotective effect of pDCs on ischemic stroke by priming Tregs.

## Materials and Methods

### Animals and Grouping

Male C57BL/6 mice weighing 21–23 g (Vital River Laboratory Animal Technology Company Limited Beijing, China) were used in this study. Animal care was carried out in accordance with guidelines approved by the Capital Medical University. All efforts were made to minimize any suffering and to reduce the number of animals used.

A total of 110 mice were included in this study. In order to detect the pDCs population change during the physiological and pathological environment, 12 mice were randomly divided into sham and MCAO group (*n* = 6 each group), from which brains, spleens, and blood were collected at 2 days after surgical procedures for flow cytometric analysis of pDCs population and the IDO1 expression level. To detect whether 120G8 is sufficient to deplete pDCs, 16 mice were randomly divided into four groups: 120G8-1d, 120G8-2d, 120G8-3d and mice without 120G8 injection (*n* = 4 each group), from which brains, spleens and blood were collected for flow cytometric analysis of pDCs. To identify the role of pDCs during the pathology of ischemic stroke, 40 mice were randomly divided into four groups: IgG+Sham (*n* = 4), 120G8+Sham (*n* = 4), IgG+MCAO (*n* = 16) and 120G8+MCAO (*n* = 16), infarct, neurological deficit and peripheral cytokines were detect at 2 days after reperfusion. In order to identify if the pDCs are still protective in the absence of Tregs, eight mice were randomly divided into two groups: anti-CD25 mAb+MCAO (*n* = 4) and anti-CD25 mAb+120G8+MCAO (*n* = 4), infarcts were detected at 2 days after reperfusion. To clarify the effect of pDCs depletion on the Tregs under physiological state and pathologic process of stroke, 24 mice were randomly divided into four groups: IgG+Sham, 120G8+Sham, IgG+MCAO and 120G8+MCAO (*n* = 6 each group), from which brains, spleens and blood were collected at 2 days after surgery for flow cytometric analysis of Tregs. In order to further identify the effect of pDCs on the Tregs induction *in vitro*, eight C57BL/6 mice were divided into sham and MCAO group (*n* = 4 each group), from which splenic pDCs were isolated. Splenic T lymphocytes from two BALB/c mice were applied to be allogeneic lymphocytes. A statistic table of experiment animals in each group was shown in Supplementary Table S1.

### Plasmacytoid Dendritic Cells Depletion

To deplete the pDCs, mice were treated with 100 μg anti-mouse pDC mAb named 120G8 (DENDRITIC, Lyon, France) or 100 μg control Ag (rat IgG, BioXcell, West Lebanon, NH, USA) intraperitoneal injection in 200 μl phosphate buffer solution (PBS) immediately before MCAO or sham procedure. The dosage was referred to as the previous studies (Wang et al., 2006; Watanabe et al., 2017). The depletion efficiency of pDCs in the brain, spleen and blood was detected with flow cytometry. In order to clarify the role of pDCs during the stroke pathology at later time points, mice were treated with 100 μg 120G8 i.p injection every 2 days after MCAO.

### Regulatory T Cells Depletion

To deplete the Tregs, mice were treated with 200 μg anti-mouse CD25 mAb (BioXcell, West Lebanon, NH, USA) intraperitoneal injection in 200 μl PBS at 3 and 1 days before MCAO. The dosage and injection time points were referred to the previous studies (Christensen et al., 2015; Clemente-Casares et al., 2016; Göschl et al., 2018). The CD3+CD4+CD25+FoxP3+ population depletion was >80% (data have not been shown).

### Transient Focal Cerebral Ischemia and Reperfusion

Immediately after injection of 120G8 or rat IgG, transient (45 min) focal cerebral ischemia was induced in mice as previously described (Gan et al., 2014; Zhao et al., 2014; Liu et al., 2018). In brief, Anesthesia was induced with 5% isoflurane and maintained with 2% isoflurane inhalation (Lunan Pharmaceutical Group Corporation, Shandong, China) in a 30% O_2_, 68.5% N_2_O mixture. Core body temperatures were maintained with a heating pad. Focal cerebral ischemia was induced for 45 min by occlusion of the right middle cerebral artery with a 6–0 MCAO suture (Doccol Corporation, Sharon, MA, USA). After 45 min of MCAO, the mice were re-anesthetized, and the occluding filament was withdrawn gently back into the common carotid artery to allow reperfusion. Exposure of the right MCA without occlusion was performed as sham surgery. The cerebral blood flow (CBF) during the surgery was measured by laser doppler perfusion monitoring with a laser Doppler probe (PeriFlux System 5000, Perimed AB, Järfälla, Sweden) interfaced to a laptop equipped with the Perisoft data acquisition software (Perimed Systems, Inc., Järfälla, Sweden) as previously described (Chen et al., 2018). The CBF data of each animal were obtained at three time points (baseline, ischemia, and 10 min after reperfusion) and presented as the percentages of baseline.

### 2,3,5-Triphenyltetrazolium Chloride Staining

For 2,3,5-triphenyl tetrazolium chloride (TTC) staining, mice were sacrificed 48 h after MCAO, and the brains were removed rapidly on ice and sliced into six coronal sections (1 mm thick). The sections were immersed in 2% TTC (Sigma–Aldrich, San Jose, CA, USA) at 37°C for 20 min and then fixed in 4% paraformaldehyde. Using the ImageJ 2× (National Institutes of Health, Bethesda, MD, USA), the infarct size with edema correction was calculated as the area of the contralateral hemisphere minus the non-infarcted area of the ipsilateral hemisphere. Data were normalized to the non-ischemic brain and expressed as a percentage.

### Immunofluorescence Staining

As previously described (Ran et al., 2018), mice were sacrificed under anesthesia at 7 days after MCAO. The brains were perfused with 0.9% saline followed by 4% paraformaldehyde in PBS (pH 7.4). The brains were harvested, fixed with 4% paraformaldehyde for 48 h, and sank in serial sucrose solutions (20% and 30%). Frozen brain tissues were cut into 30 μm sections. All samples were attached to slides and stored in the slide box at −20°C. For immunohistochemistry staining, sections were washed in PBST (3 × 10 min, pH 7.4), and incubated with 10% serum for 30 min at room temperature. The sections were then incubated with primary antibody (rabbit anti-MAP-2 antibody, 1:100, 17490-1-AP, Proteintech Group Inc., Wuhan, China) overnight at 4°C. The sections were washed with PBST (3 × 10 min) and incubated with secondary antibodies (conjugated with Alexa Fluor 488 goat anti-rabbit IgG, 1:100, P03S05, Gene protein Link, Beijing, Chian) for 2 h at room temperature. After the final wash with PBST (3 × 10 min), sections were counterstained with the Fluorescent Mounting Medium with DAPI (ZLI-9557, Zsbio Commerce Store, Beijing, Chian) and covered with coverslips. All samples were examined under a laser scanning confocal microscope.

### Neurological Deficit Assessment

Neurological deficit assessment was performed by investigators blinded to the control and MCAO groups, as described elsewhere (Bederson et al., 1986; Gan et al., 2014). The mice were examined and assessed at 0.5, 24, and 48 h after brain reperfusion. The scoring system was used as follows: 0 = no deficit, 1 = failure to extend left forepaw, 2 = decreased grip strength of left forepaw, 3 = circling to left by pulling the tail, and 4 = spontaneous circling. The animals showing no obvious sign of neurological deficits (neurological score of less than 2) were excluded.

### Leukocyte Harvest From Brain, Spleen, and Blood

At 2 day after reperfusion, the leukocytes from the brain, spleen and blood were harvested. For brain cell isolation, the brains were cut into small pieces and dissociated into single-cell suspensions with the Adult Brain Dissociation Kit (130-107-677, Miltenyi Biotec, Bergisch Gladbach, Germany) on the gentleMACS Octo Dissociator with Heaters (130-096-427, Miltenyi Biotec, Bergisch Gladbach, Germany) running program 37C_ABDK_1, according to the manufacturer’ s instructions. Spleen single-cell suspensions were obtained by grinding followed by filtration through a nylon mesh. Blood was taken from the heart with a 1 ml syringe and rapidly transferred to heparin saline anticoagulant tubes. For each mouse, 0.5 ml blood was harvested. For leukocyte retrieval, the samples from the spleen and blood were centrifuged at 3,000 rpm for 4 min. The pellets were treated with the red blood cell lysis buffer (Beyotime Biotechnology Company Limited, Jiangsu, China) and washed twice with PBS (HyClone Laboratories Inc., Logan, UT, USA), to remove red cells. The leukocytes were analyzed by flow cytometry.

### Flow Cytometry Analysis

Leukocyte classification by phenotypic analysis (the surface expression of antigen markers) was performed by flow cytometry. Leukocytes were resuspended in PBS at a concentration of 2 × 10^5^/ml and stained with the fluorochrome-conjugated antibodies in darkness for 30 min at room temperature. All antibodies were purchased from Biolegend (San Diego, CA, USA), including fluorescein isothiocyanate (FITC) anti-mouse CD3 (100204), FITC anti-mouse CD11c (117306), peridinin chlorophyl protein (PerCP) anti-mouse CD4 (100432), PerCP/Cy5.5 anti-mouse PDCA-1 (127021), phycoerythrin (PE) anti-mouse B220 (103208), allophycocyanin (APC) anti-mouse CD25 (102012), phycoerythrin (PE) anti-mouse FoxP3 (202307), True-Nuclear™ Transcription Factor Buffer Set (424401) and Alexz Fluor^®^ 647 anti-IDO1(654003). Appropriate isotype-matched immunoglobulins were used as negative controls. Cells were analyzed on the BD C6 flow cytometer with FlowJo software (Becton Dickinson, San Jose, CA, USA). The targeted populations were gated on the scatter plots of forward scatter (FSC-A) and side scatter (SSC-A), excluding debris and cell aggregates; pDCs and Tregs were further gated based on their expression of specific markers. We defined the CD11c^int^PDCA1^+^ population as pDCs and CD3^+^CD4^+^CD25^+^FoxP3^+^ population as Tregs (Lippens et al., 2016).

### pDCs and Pan T Cells Isolation

For pDCs isolation, the spleens were dissociated into single-cell suspensions with spleen dissociation kit (130-095-926, Miltenyi Biotec, Bergisch Gladbach, Germany) on the gentleMACS Octo Dissociator with Heaters (130-096-427, Miltenyi Biotec, Bergisch Gladbach, Germany) running program 37C_m_SDK_1. For pan T cells isolation, the spleen single-cell suspensions were obtained by grinding followed by filtration through a nylon mesh. pDCs and pan T cells were purified from splenocytes using pDCs isolation kit (130-107-093, Miltenyi Biotec, Bergisch Gladbach, Germany) and pan T cell isolation kit II (130-095-130, Miltenyi Biotec, Bergisch Gladbach, Germany) respectively, according to the manufacturer’s instructions.

### Mixed Lymphocyte Reaction

MLR proceeded as previously described (Chen et al., 2014). T lymphocytes (1 × 10^6^/ml) from BALB/c mice were stained with CFSE and then co-cultured with pDCs from C57BL/6 (1 × 10^5^/ml) after mitomycin C treatment (BioVision, Milpitas, CA, USA). After 5 days, harvested cells were analyzed for phenotyping and T-cell proliferation was evaluated by flow cytometry.

### ELISA Analysis

Concentrations of IL-6, IL-10, TNF-α and IL-1β in plasma were measured with commercially available enzyme-linked immunosorbent assay (ELISA) kits from Dakewe Biotech company (Shenzhen, China) according to the manufacturer’ s instructions, as previously described (Chen et al., 2014; Yang et al., 2016).

### Western Blot

Total protein was isolated from pDCs with RIPA lysis buffer (Beyotime Biotechnology, Shanghai, China) containing 1 mM PMSF (Beyotime Biotechnology, Shanghai, China). Between 50 and 60 μg protein per sample was loaded on a 10% SDS-PAGE gel and transferred onto nitrocellulose membranes. The membranes were incubated with primary antibodies (Proteintech Group Inc., Wuhan, China) as indicated overnight and then incubated with horseradish peroxidase-conjugated secondary antibody for 1 h. The blots were developed with an enhanced chemiluminescence detection system (Beijing Sage creation) and the bands were scanned. The densitometry analysis was performed with the software ImageJ 2 × (National Institutes of Health, Bethesda, MD, USA). The intensity of protein bands was quantified and shown as the ratio to sham after normalization by GAPDH.

### Statistical Analysis

All results were represented as the means ± SEM. Differences between two groups were evaluated for statistical significance using a student’s *t*-test. Differences between three or more groups were evaluated for statistical significance using one-way ANOVA followed by Bonferroni *post hoc* test. The normality of the distribution and homogeneity of variance were assessed by the *F*-test or Bartlett’s test before the student’s *t*-test and the ANOVA, respectively. A two-tailed *p*-value of <0.05 was considered statistically significant.

## Results

### MCAO Increases the Number of pDCs in the Brain

**Figure 1:**
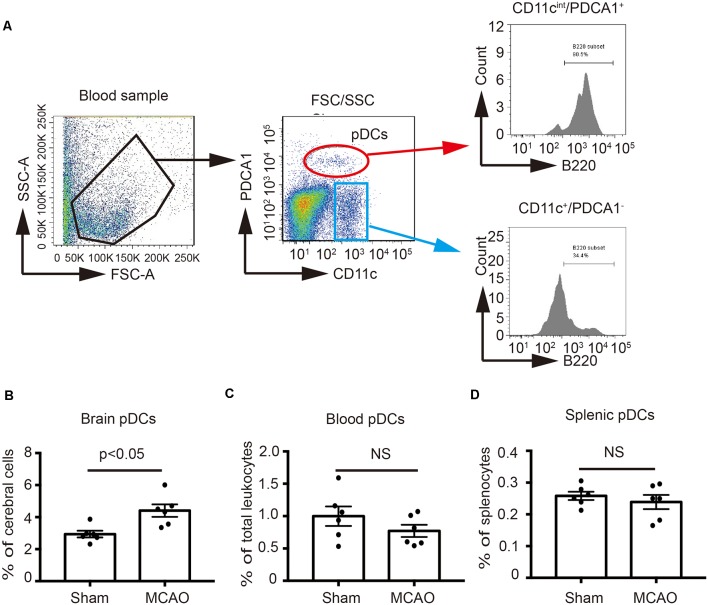
Middle cerebral artery occlusion (MCAO) increased the number of plasmacytoid dendritic cells (pDCs) in the brain. Brain ischemia was induced by 45 min MCAO. Sham-operated animals underwent surgical exposure of the right middle cerebral artery without occlusion. **(A)** The gating strategy to identify the pDCs in the blood. The pDCs population (CD11c^int^PDCA1^+^B220^+^) in the **(B)** brain, **(C)** spleen and **(D)** blood was analyzed with Flow cytometry. Data are expressed as means ± SEM for *n* = 6 mice per group. The scatter plots represent independent samples. NS: no significance.

In order to identify the effect of MCAO on the pDCs, the brains, spleens and blood samples were collected at 2 days after MCAO, and then pDCs were analyzed by flow cytometry. As shown in Figure 1A, we defined the CD11c^int^PDCA1^+^ population as pDCs according to the previous studies (Lippens et al., 2016; Segura, 2016) which highly expressed B220, while the CD11c^+^PDCA1^−^ population have lower B220 expression. B220 is a unique surface marker of pDCs (Segura, 2016). Moreover, the percentages of brain pDCs were significantly increased 2 days after MCAO (4.58% vs. 2.94% in Figure 1B). However, MCAO exerted minimal effects on the percentages of blood and splenic pDCs (0.82% vs. 0.94% in Figure 1C, 0.25% vs. 0.26% in Figure 1D, respectively). These results suggest that pDCs participate in the pathology of ischemic stroke-induced brain injury.

### Depletion of pDCs Exacerbates MCAO-Induced Brain Injury in Mice

**Figure 2:**
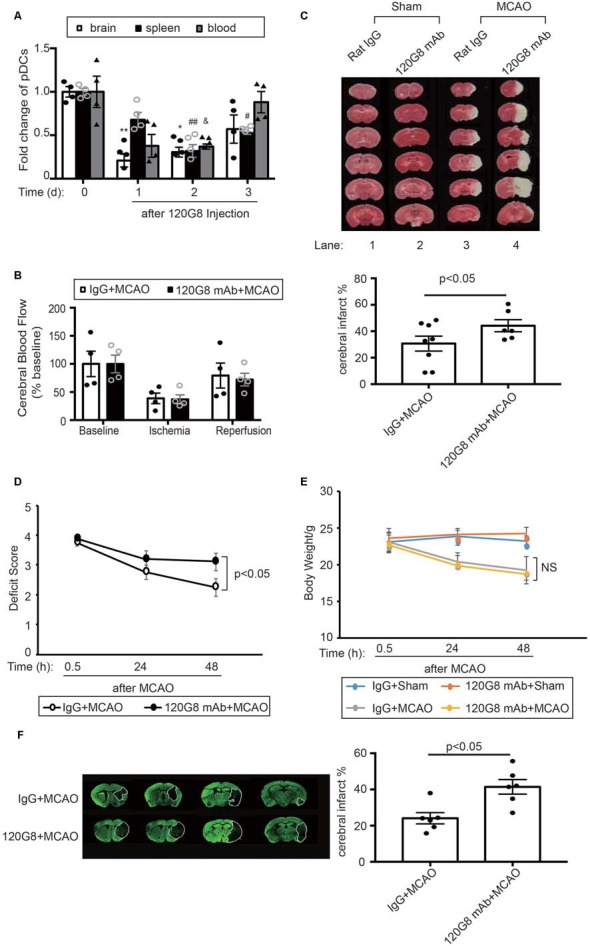
Depletion of pDCs exacerbates MCAO-induced brain injury. One-hundred micrograms 120G8 mAb or rat IgG i.p.injection was conducted immediately before MCAO or sham procedure in mice. **(A)** The deletion efficiency of pDCs in the brain, spleen and blood at different time points. **(B)** Cerebral blood flow (CBF) during the MCAO was measured at three time points: baseline, ischemia, and reperfusion. Data are normalized to baseline and expressed as percentages. **(C)** Representative triphenyl tetrazolium chloride (TTC) images of animals from each group and corresponding statistical analysis. **(D)** Neurological deficit assessment was performed with the longa scoring system during the 2 days after MCAO. **(E)** Bodyweight of all mice from each group was measured during the 2 days after MCAO. **(F)** Representative images of MAP-2-stained coronal brain sections at 7 days after MCAO and corresponding statistical analysis. Data are expressed as means ± SEM for *n* = 4–10 mice per group. *p < 0.05, **p < 0.01 vs. cerebral pDCs without 120G8 i.p. injection. ^#^*p* < 0.05, ^##^*p* < 0.01 vs. splenic pDCs without 120G8 i.p. injection. ^&^*p* < 0.05 vs. pDCs in blood without 120G8 i.p. injection. The scatter plots represent independent samples. NS: no significance.

In order to identify the role of pDCs during the pathology of ischemic stroke. We depleted pDCs with anti-pDCs Ag mAb (120G8) prior to the MCAO procedure in mice. As shown in Figure 2, the pDCs populations in the brain, spleen and blood were simultaneously reduced to about 30% of baseline with significance at 2 days after 120G8 treatment (Figure 2A). The CBF, detected during the MCAO surgery, was reduced to less than 40% of baseline during ischemia and reestablished to 70% of baseline after reperfusion without significant differences between groups (Figure 2B). At 2 days after reperfusion, mice subjected to 120G8 injection demonstrated significant increases in infarct sizes (lane4 vs. lane3 in Figure 2C) and deficit scores (Figure 2D) compared to animals with Rat IgG injection at 48 h after MCAO, whereas the body weights remained unchanged between IgG and 120G8 group followed by sham or MCAO (Figure 2E). In order to clarify the role of pDCs during the stroke pathology at a later time point, the infarcts were detected at 7 days with MAP-2 stain. As shown in Figure 2F, mice subjected to 120G8 injection demonstrated significant increases in infarct sizes. The above data demonstrate that pDCs could protect against MCAO-induced brain injury.

### Depletion of pDCs Accelerates the Peripheral Pro-inflammatory Response Followed by MCAO

**Figure 3:**
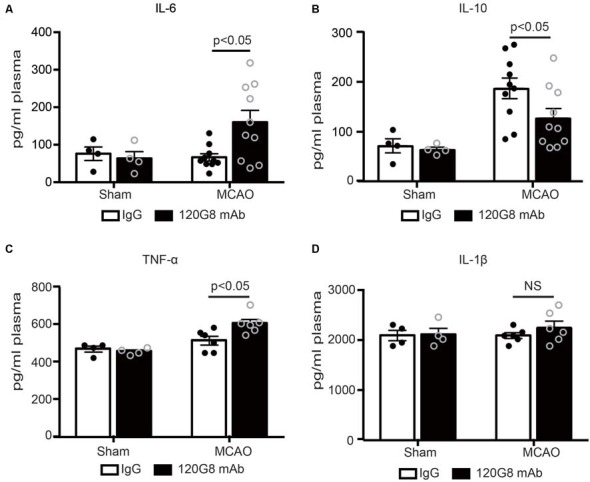
Depletion of pDCs accelerates the peripheral pro-inflammatory response followed by MCAO. One-hundred micrograms 120G8 mAb or rat IgG i.p injection was conducted immediately before MCAO or sham procedure in mice. Cytokines in the plasma from each group were detected with ELISA. **(A)** Statistical analysis of IL-6 expression. **(B)** Statistical analysis of IL-10 expression. **(C)** Statistical analysis of TNF-α expression. **(D)** Statistical analysis of IL-1β expression. Data are expressed as means ± SEM for *n* = 4–10 mice per group. The scatter plots represent independent samples. NS: no significance.

Moreover, pDCs depletion also altered corresponding circulating cytokine profiles after stroke. As shown in Figure 3, 120G8 exerted minimal effects on the cytokine levels under the physiological condition. However, pDCs depletion could induce the pro-inflammatory response under the pathological condition of stroke, including the significantly elevated IL-6, TNF-α concentration (160.17 pg/ml vs. 66 pg/ml in Figure 3A; 605.50 pg/ml vs. 514.37 pg/ml in Figure 3C), and reduced IL-10 level (126.36 pg/ml vs. 185.94 pg/ml in Figure 3B) in plasma of MCAO mice, while the IL-1β concentration had no notable changes (2,258 pg/ml vs. 2,093 pg/ml in Figure 3D). The above data demonstrated that pDCs may protect against ischemic stroke-induced brain injury through modulating systemic anti-inflammatory response.

### Depletion of pDCs Decreases the Tregs Population in the Periphery and Brain Followed by MCAO

**Figure 4:**
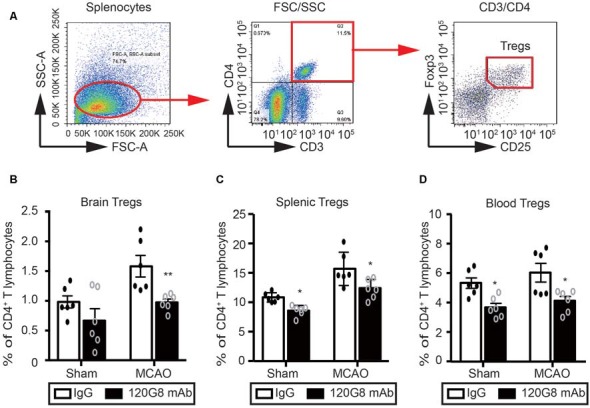
Depletion of pDCs decreases the Tregs population in the periphery and brain followed by MCAO. One-hundred micrograms 120G8 mAb or rat IgG i.p.injection was conducted immediately before MCAO or sham procedure in mice. The brains, spleens and blood samples were collected 2 days after MCAO or sham procedures. **(A)** The gating strategy to identify the splenic Tregs. The Tregs population (CD3^+^CD4^+^CD25^+^Foxp3^+^) in the **(B)** brain, **(C)** spleen and (**D)** blood was analyzed with Flow cytometry. Data are expressed as means ± SEM for *n* = 6 mice per group. *p ≤ 0.05, **p ≤ 0.01 vs. IgG group. The scatter plots represent independent samples.

Considering the protective role and anti-inflammatory property of pDCs in the pathology of ischemic stroke, we detected the effect of pDCs depletion on the Tregs after MCAO with flow cytometry. The gating strategy of Tregs was shown in Figure 4A. Under physiological condition, 120G8 induced the downtrend of cerebral Tregs (0.65% vs. 0.92% in Figure 4B) and obviously reduced the splenic and blood Tregs (8.92% vs. 11.39% in Figure 4C; 3.60% vs. 5.2% in Figure 4D). Moreover, pDCs depletion significantly reduced the Tregs in the brain, spleen and blood (0.92% vs. 1.55% in Figure 4B; 12.24% vs. 15.7% in Figure 4C; 4.11% vs. 6.03% in Figure 4D) under the pathologic state of stroke. The findings here suggested that the protective role and anti-inflammatory property of pDCs in the pathology of ischemic stroke may be related to Tregs.

### The pDCs Accelerate the Tregs Induction Under the Pathologic State of Stroke

**Figure 5:**
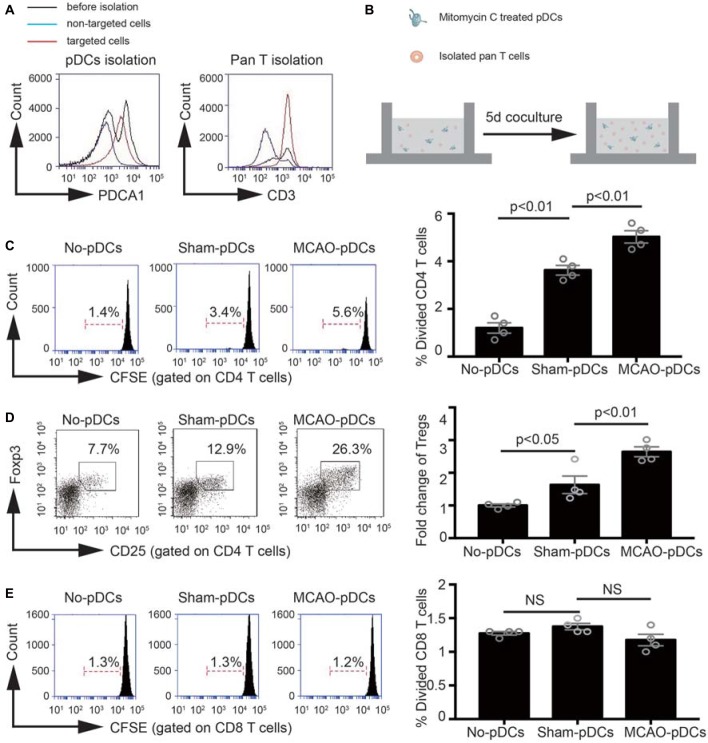
The pDCs accelerates the Tregs induction under the pathologic state of stroke. Brain ischemia was induced by 45 min MCAO. Sham-operated animals underwent surgical exposure of the right middle cerebral artery without occlusion. The splenic pDCs and allogeneic T lymphocytes were purified through magnetic isolation and cocultured by 1:10 for 5 days *in vitro*. The phenotype and proliferation of T lymphocytes were analyzed with flow cytometry. **(A)** The pDCs and pan T cells were isolated from a mouse spleen cell suspension by using the magnetic isolation kit. **(B)** Schematic illustration of mixed lymphocytes reaction. **(C)** *In vitro* proliferation of CD4 T lymphocytes, assays were performed with the fluorescent dye CFSE and analyzed by flow cytometry. **(D)** At day 5 of coculture, cells were harvested and the Tregs (CD25+ Foxp3+ population gated on the CD4 T cells) were analyzed by flow cytometry. Indicated numbers are the mean percentages of gated cells. **(E)** *In vitro* proliferation of CD8 T lymphocytes, assays were performed with the fluorescent dye CFSE and analyzed by flow cytometry. The loss of CFSE fluorescence reflects cellular division. Indicated numbers are the percentages of dividing cells. Data are expressed as means ± SEM for *n* = 4 mice per group. The scatter plots represent independent samples. NS: no significance.

In order to further identify the effect of pDCs on the Tregs induction under the pathologic state of stroke. We detect the immunostimulatory capacity of pDCs in a MLR *in vitro*. As shown in Figures 5A,B, pDCs and T lymphocytes were purified through magnetic isolation and cocultured for 5 days. The phenotype and proliferation of T lymphocytes were analyzed with flow cytometry. CD4 and CD8 T lymphocytes alone had low proliferation ability (1.4% in Figure 5C, 1.3% in Figure 5E; No-pDCs). The pDCs markedly increased CD4 T lymphocytes proliferation (3.4% vs. 1.4% in Figure 5C), while exerted no effect on the CD8 T lymphocytes (1.3% vs. 1.3% in Figure 5E). In addition, pDCs derived from MCAO mice significantly promoted CD4 T lymphocytes proliferation compared with the sham-pDCs (5.6% vs. 3.4% in Figure 5C), while had no effect on the CD8 T lymphocytes (1.2% vs. 1.3% in Figure 5E). Meanwhile, the Tregs population gated on the CD4 T lymphocytes was detected with flow cytometry. As shown in Figure 5D, pDCs from sham mice promoted the Tregs induction (12.9% vs. 7.7% in Figure 5D), and pDCs from MCAO mice further promoted the Tregs proliferation (26.3% vs. 12.9% in Figure 5D). These results implied the pDCs could prime the Tregs under a physiologic state and accelerates the Tregs induction under the pathologic state of stroke.

### MCAO Increased the IDO1 Expression on pDCs

**Figure 6:**
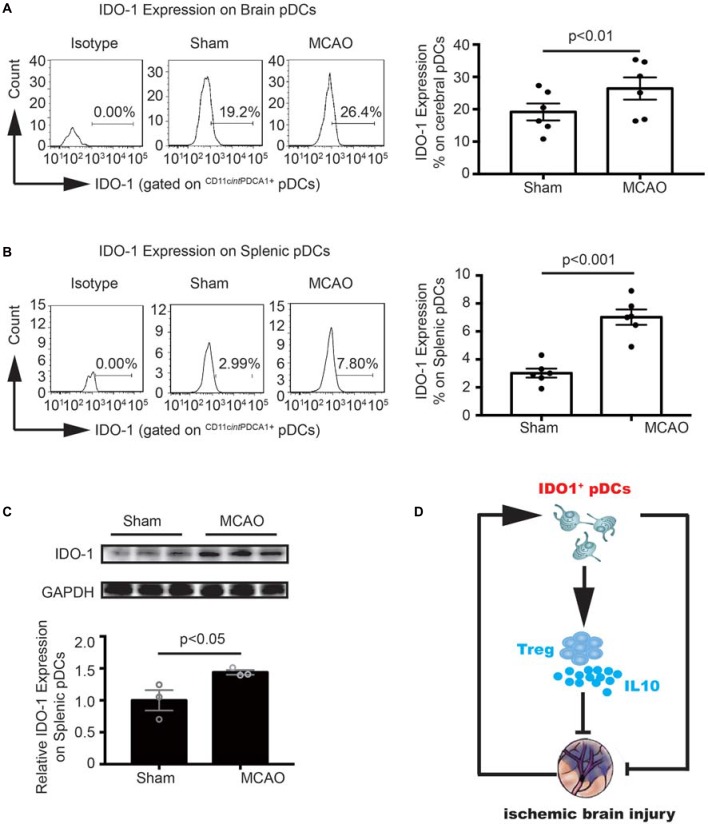
MCAO increased the Indoleamine 2,3-dioxygenase 1 (IDO1 expression on pDCs. Brain ischemia was induced by 45 min MCAO. Sham-operated animals underwent surgical exposure of the right middle cerebral artery without occlusion. The brain and spleen samples were collected at 2 days after MCAO. IDO1 expressions on pDCs from the brain **(A)** and spleen **(B)** were analyzed with Flow cytometry. Isotype controls (left), representative flow cytometric images from each group (middle) and corresponding statistical analysis (right) were shown. Indicated numbers are the mean percentages of IDO1^+^ pDCs. Data are expressed as means ± SEM for *n* = 6 mice per group. **(C)** The protein levels of IDO1 were detected with Western blot analysis. Representative Western blots were shown (above). The intensity of protein bands was quantified and shown as the ratio to sham after normalization by GAPDH (below). The results are expressed as mean ± SEM for three independent experiments. The scatter plots represent independent samples. **(D)** Schematic illustration of IDO1^+^ pDCs-regulated protection against MCAO-induced brain injury and its association with activation of Tregs.

To further explore the molecular mechanism underlying the pDCs induced Tregs response. We detect the IDO1 expression on the cerebral and splenic pDCs with flow cytometry and western blot. As illustrated in Figure 6, the IDO1 expression on the cerebral and splenic pDCs were significantly elevated 2 days after MCAO (26.4% vs. 19.2% in Figure 6A, 7.8% vs. 2.99% in Figure 6B). Moreover, the protein level of IDO1 on the splenic pDCs from MCAO mice was obviously elevated (Figure 6C). Together, the results in this part suggested that the increased IDO1 expression on pDCs is critical for Tregs priming under the pathology of stroke. Finally, as shown in Figure 6D, pDCs protect against stroke by priming regulatory T cells accompanied with the increased IDO1 expression.

## Discussion

Ischemic stroke results in a rapid systemic inflammatory response, which exacerbates the initial infarct (Huang et al., 2006; Ross et al., 2007). Modulation of tolerogenic immune and anti-inflammatory responses represents an important target for improving clinical outcomes after stroke (Becker, 2010). Cerebral hypoxia and ischemia cause necrotic brain cell death and promote the release of danger signals from the injured tissue (Chamorro et al., 2012). The antigen-presentation cells (APCs) are equipped with danger signal sensors and orientate the immune and inflammatory responses. DCs are professional APCs that constantly survey the environment. Our study focused on the effect of pDCs, a subset of DCs, which are able to induce immune tolerance in the pathology of ischemic stroke and the underlying mechanism.

pDCs are a rare type of immune cell which circulate in the blood and is found in peripheral lymphoid organs. They develop from bone marrow hematopoietic stem cells and constitute less than 0.4% of splenocytes and peripheral blood mononuclear cells (Tversky et al., 2008; Yun et al., 2016), which is consistent with our data about splenic pDCs percentages. While we found that the frequencies of pDCs in peripheral blood were slightly higher than the physiological level, which may be related to the activation of the systemic immune response after sham or MCAO procedure. Moreover, we found that the pDCs in the brain were significantly increased after MCAO and suppressing pDCs with 120G8 exacerbated the brain injury and peripheral pro-inflammation after MCAO, which inferred that the pDCs may protect against the ischemic stroke-induced brain injury through modulating tolerogenic immune and anti-inflammatory responses. The pDCs have multifaceted biology. On the one hand, pDCs specialize in the production of type I interferons (IFNs) to promote antiviral immune responses and participate in the pathogenesis of autoimmune diseases (Isaksson et al., 2009; Alculumbre et al., 2019). On the other hand, pDCs are also reported to have a role in peripheral and central tolerance (Hadeiba et al., 2008, 2012). The tolerogenic property of pDCs is mostly associated with the induction of Tregs (Puccetti and Grohmann, 2007; Swiecki and Colonna, 2015).

Tregs are a subpopulation of T cells that represents 5–10% of circulating CD4^+^ T cells (Battaglia et al., 2006). They are important for the maintenance of immune homeostasis and the suppression of excessive immune responses. In stroke patients, the number of circulating Tregs decreases dramatically soon after stroke (Urra et al., 2009; Yan et al., 2009). Using carboxyfluorescein diacetate succinimidyl ester (CFSE) to track splenocyte migration following MCAO, it has been shown that splenocytes including lymphocytes can enter the systemic circulation and migrate into the brain (Seifert et al., 2012). In our study, we simultaneously detected the Tregs population in the brain, spleen and blood. We found that MCAO induced the uptrend of the cerebral, splenic and blood Tregs population, which may be related to the exacerbated systemic inflammation (including pro- and anti-inflammatory response) post-stroke. Moreover, depletion of pDCs using 120G8 could significantly decrease the Tregs in the brain, spleen and blood under the pathologic state of stroke, demonstrating the anti-inflammatory property of pDCs in the pathology of ischemic stroke may be related to Tregs.

In order to further identify the direct relation between the pDCs and Tregs. The immunostimulatory capacity of pDCs was detected. Interestingly, under the physiological state, the pDCs had the ability to stimulate the CD4 T lymphocytes and Tregs proliferation, and the immunostimulatory capacity on the CD4 T lymphocytes and Tregs was strengthened under the pathological state of stroke. Our data implied the protective effect of pDCs against stroke can be manifested by priming the Tregs characterized by immune tolerance.

Mechanically, accumulating studies have implicated IDO1 expression on pDCs was necessary to confer suppressive function to Tregs in many diseases such as melanoma, experimental autoimmune encephalomyelitis (EAE), rheumatoid arthritis and atherosclerosis (Kavousanaki et al., 2010; Chevolet et al., 2015; Lippens et al., 2016; Yun et al., 2016). Following cerebral ischemia, levels of IDO1 in infarct and penumbra regions are reported to increase at 24 h after stroke (Jackman et al., 2011). The activity of IDO1 has been reported to be elevated in stroke patients (Darlington et al., 2007). In this study, we found the increased IDO1 expression on the splenic pDCs, which may be related to the proliferation of local Tregs. Although there are no significant changes in IDO1 expression on the cerebral pDCs, the IDO1 levels are higher than that on splenic pDCs. Collectively, the above data illustrate that under the pathology of cerebral ischemia, the induction of Tregs characterized by immunologic tolerance is dependent on the elevated IDO1 expression of pDCs. Although further studies are warranted, all data here might highlight a new and effective entry point for the prevention of ischemic brain injury by targeting IDO1^+^ pDCs.

In most studies, Tregs were found to proliferate and show protective effects at 7 days post MCAO or even later, and only a very low number of Tregs were observed within the first week after transient ischemia (Gelderblom et al., 2009; Stubbe et al., 2013; Liesz and Kleinschnitz, 2016), which implied that it seems to be questionable if the protective effects of pDCs are directly mediated *via* Tregs. In our study, we found that under physiological condition, 120G8 induced the downtrend of cerebral Tregs and obviously reduced the splenic and blood Tregs, which inferred that the pDCs depletion oriented the systemic pro-inflammation and pDCs protecting against ischemic brain injury may be realized by maintaining the systemic immune homeostasis, which is consistent with the notion that pDCs are in association with Tregs, serving as a guide for immunotherapeutic options in acute liver failure (Koda et al., 2019).

Moreover, we identify the depletion of pDCs deteriorated the infarct at a later time, suggesting the persistent protective role of pDCs. It is interesting to study if the pDCs are still protective in the absence of Tregs. As shown in Supplementary Figure S1, Tregs depletion inhibited the pDCs depletion-induced increased infarct, which implies pDCs lost protective effect in the absence of Tregs, even worsen the outcome of stroke. This phenomenon deserves further study to explore the multifaced function of pDCs in the pathology of stroke.

## Data Availability Statement

The datasets generated for this study are available on request to the corresponding author.

## Ethics Statement

The animal study was reviewed and approved by The Capital Medical University.

## Author Contributions

CC designed and performed the experiments, collected and analyzed data, and drafted the manuscript. ZC performed all the animal experiments, including MCAO surgery, animal sacrifice, and sampling. LC contributed to the experimental design and manuscript. GX supervised the research group and the study.

## Conflict of Interest

The authors declare that the research was conducted in the absence of any commercial or financial relationships that could be construed as a potential conflict of interest.

## Supplementary Material

FIGURE S1 Plasmacytoid dendritic cells (pDCs) lost protective effect in the absence of Tregs. Two-hundred micrograms anti-mouse CD25 mAb i.p.injection was conducted at −3 and −1 day, following by 100 μg 120G8 mAb immediately before middle cerebral artery occlusion (MCAO) or not in mice. Representative TTC images of animals from each group and corresponding statistical analysis. Data are expressed as means ± SEM for *n* = 4 mice per group. The scatter plots represent independent samples.

TABLE S1 Statistics of experiment animals in each group. The animals showing no obvious sign of neurological deficits (neurological deficit score less than 2) were excluded.
