# Supplementary figures and images for: Plasmacytoid Dendritic Cells Protect Against Middle Cerebral Artery Occlusion Induced Brain Injury by Priming Regulatory T Cells
Source: Front Cell Neurosci. 2020 Jan 31;14:8. doi: 10.3389/fncel.2020.00008 (PMC7006436; doi:10.3389/fncel.2020.00008)

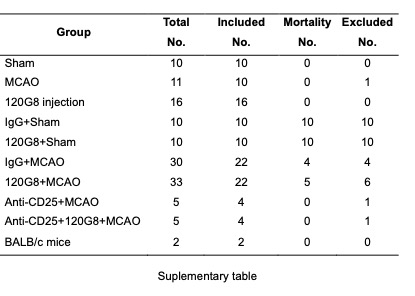

Supplement: FIGURE S1 — Plasmacytoid dendritic cells (pDCs) lost protective effect in the absence of Tregs. Two-hundred micrograms anti-mouse CD25 mAb i.p.injection was conducted at −3 and −1 day, following by 100 μg 120G8 mAb immediately before middle cerebral artery occlusion (MCAO) or not in mice. Representative TTC images of animals from each group and corresponding statistical analysis. Data are expressed as means ± SEM for n = 4 mice per group. The scatter plots represent independent samples. [file Image_1.JPEG]

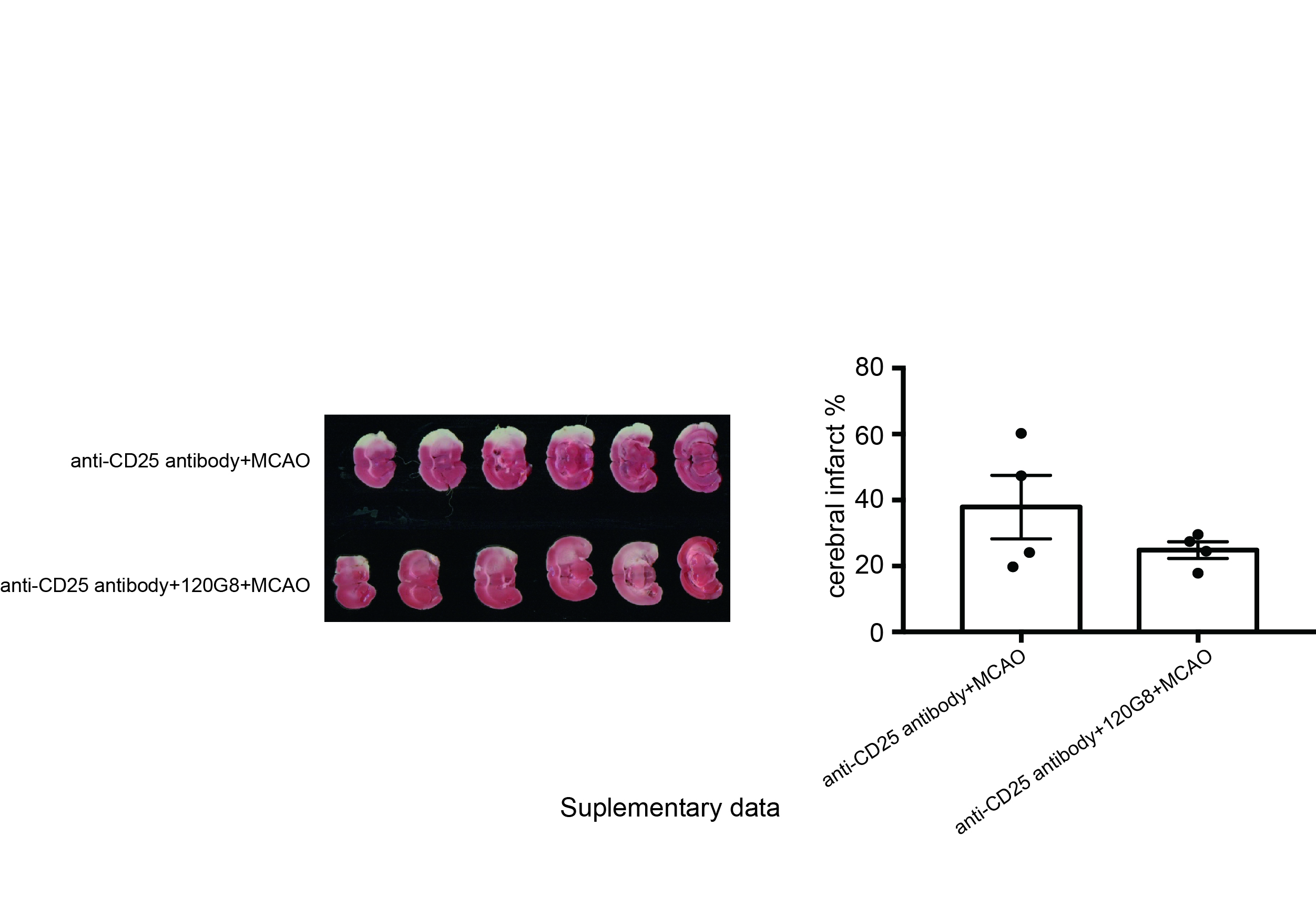

Supplement: TABLE S1 — Statistics of experiment animals in each group. The animals showing no obvious sign of neurological deficits (neurological deficit score less than 2) were excluded. [file Image_2.JPEG]
